# Isolation of folate-producing probiotic candidates and their effects on homocysteine metabolism and gut microbiota composition

**DOI:** 10.3389/fnut.2026.1845105

**Published:** 2026-08-20

**Authors:** Mingfang Pan, Changming Ye, Yun Song, Minqing Tian, Runming Wang, Ping Chen

**Affiliations:** 1 Institute of Biopharmaceutical and Health Engineering, Shenzhen International Graduate School, Tsinghua University, Shenzhen, China; 2 Shenzhen Evergreen Medical Institute, Shenzhen, China; 3 Inspection and Testing Center, Key Laboratory of Cancer FSMP for State Market Regulation, Shenzhen, China; 4 AUSA Research Institute, Shenzhen AUSA Pharmed Co., Ltd., Shenzhen, China; 5 Precision Nutrition Innovation & Transformation Public Service Platform, Shenzhen, China

**Keywords:** B vitamin, folate, gut microbiota, homocysteine, microbial cross-feeding, micronutrient, probiotic

## Abstract

**Background:**

Folate deficiency is a global nutritional problem associated with multiple adverse health outcomes, including impaired one-carbon metabolism and elevated homocysteine levels (hyperhomocysteinemia). Gut microbiota-mediated folate biosynthesis has emerged as a promising strategy for improving the host’s folate status. This study aimed to isolate folate-producing probiotic strains, clarify their folate synthesis mechanisms, and evaluate their regulatory effects on folate metabolism and gut microbiota.

**Methods:**

High-throughput cultivation and screening were performed to isolate folate-producing candidate probiotics. Whole-genome sequencing analysis, pathway reconstruction, and metabolite profiling in fermented milk were performed to explore folate biosynthesis pathways and microbial cross-feeding interactions. A folate-deficient mouse model was established to evaluate the effects of a candidate probiotic cocktail on serum folate, homocysteine (Hcy) levels, and gut microbiota composition using quantitative PCR (qPCR) and 16S rRNA gene sequencing.

**Results:**

High-throughput screening identified 8 high-folate-producing candidate probiotic strains, including *Lactiplantibacillus plantarum* and *Heyndrickxia coagulans*, from over 1,000 isolates. Genomic analysis revealed that most commonly used probiotics lacked para-aminobenzoic acid (pABA) biosynthesis genes but retained downstream modules, suggesting a reliance on cross-feeding with pABA-producing gut commensals such as Bacteroides. Metabolite profiling of fermented milk demonstrated that selected strains significantly increased bioactive 5-methyltetrahydrofolate (5-MeTHF) and tetrahydrofolate levels. *In vivo*, only a high-dose candidate probiotic cocktail significantly elevated serum folate (*p* < 0.05) and reduced homocysteine levels (*p* < 0.05) in deficient mice. Fecal qPCR confirmed dose-dependent transient persistence of the administered bacterial species. Consistent with the qPCR data, 16S rRNA gene sequences demonstrated significant enrichment of these administered species observed in the high-dose group. Furthermore, beta-diversity analysis found that high-dose candidate probiotic supplementation promoted a shift in the gut microbiota composition toward a normal profile, partially mitigating the dysbiosis induced by the folate-deficient diet. This effect was accompanied by a significant enrichment of potential short-chain fatty acid producers (e.g., *Lachnospiraceae* and *Oscillospiraceae*) and the depletion of potential opportunistic pathogens.

**Conclusion:**

This study screened high-folate-producing candidate probiotic strains and demonstrated their ability to synthesize the active form of 5-MeTHF. Moreover, folate-producing candidate probiotic cocktail treatment significantly improved folate status and Hcy metabolism and modulated the gut microbiota by enriching potential beneficial bacterial taxa. These findings suggested that folate-producing probiotics may serve as a promising microbiota-based strategy to improve folate availability and homocysteine metabolism.

## 1 Introduction

Folate is an essential water-soluble B vitamin that plays a pivotal role in one-carbon metabolism, including DNA synthesis, methylation reactions, and amino acid metabolism (1). Inadequate folate availability is closely associated with elevated plasma homocysteine levels (hyperhomocysteinemia), a recognized risk factor for cardiovascular diseases, neurodegenerative disorders, and metabolic syndrome (2). Although folic acid supplementation has been widely adopted to prevent folate deficiency, increasing evidence suggests that excessive intake of synthetic folic acid may lead to unmetabolized folic acid accumulation and potential adverse health effects, highlighting the need for safer and more physiologically relevant alternatives (3).

Although dietary intake remains the primary determinant of folate and vitamin B12 status, emerging evidence suggests that the gut microbiota may play a contributory role in regulating host folate status (4). Specific commensal bacteria, particularly lactic acid bacteria and bifidobacteria, endogenously synthesize biologically active folate derivatives, including 5-methyltetrahydrofolate (5-MeTHF), the predominant circulating form of folate in humans (5, 6). Unlike synthetic folic acid, which requires rate-limiting reduction by dihydrofolate reductase (DHFR) to become metabolically active, folates synthesized by bacteria are primarily fully reduced THFs with methyl, methylene, or formyl groups. Although these bacterial folates are often polyglutamylated and require deconjugation to monoglutamate by intestinal brush-border enzymes prior to absorption, they bypass the DHFR bottleneck, which is particularly attractive for patients with inactivated single-nucleotide polymorphisms (SNPs) in DHFR (5, 7). Thus, folate-producing probiotics represent a promising strategy for improving folate nutrition and regulating homocysteine metabolism through microbiota-mediated mechanisms (8, 9).

Previous studies have reported the isolation of folate-producing probiotic strains from both traditional fermented foods (10–13) and the human gut (9, 14, 15), yet most studies quantify total folate production using microbiological assays or liquid chromatography, without subsequent mass spectrometry validation of bioactive folate profiles. This methodological limitation likely reflects the inherent instability of 5-methyltetrahydrofolate (5-MeTHF), the primary bioactive folate, which readily oxidizes to derivatives (16). Furthermore, the therapeutic efficacy of these probiotics (as single strains or consortia) in enhancing host folate bioavailability, modulating homocysteine metabolism, and remodeling gut microbial ecology has not been systematically investigated in animal models or human trials.

In the present study, we isolated and identified folate-producing candidate probiotic strains from traditional fermented foods and human gut microbiota. Their folate-producing capacities were evaluated by quantifying total folate and 5-MeTHF levels using microbiological assays and LC–MS/MS analysis, respectively. Furthermore, candidate probiotic cocktails composed of selected high-folate-producing strains were administered to mice to investigate their effects on homocysteine metabolism and gut microbiota composition. This research provides novel insights into the functional role of folate-producing probiotics as a natural and microbiota-targeted approach for modulating host homocysteine metabolism and improving metabolic health.

## 2 Materials and methods

### 2.1 Isolation and identification of candidate probiotic strains

Potential folate-producing strains were isolated from traditional cow’s milk kefir from Tibet and from human stool using an established high-throughput workflow adapted from previously reported methods (11, 17). Approximately 1 g of sample was suspended in 10 mL of 1 × PBS, and a series of dilutions was prepared and cultured on MRS agar plates supplemented with 2% CaCO_3_ (pH 6.5). The plates were then incubated anaerobically for 48 h at 37 °C. The bacterial colonies that formed clear zones around them from dissolving CaCO_3_ were randomly isolated. Then each isolated strain was anaerobically incubated in MRS broth at 37 °C for 24 h without shaking. Different cell cultures were collected, washed, and resuspended in sterilized 1 × PBS. Subsequently, the obtained suspensions were inoculated into folic acid assay broth (BioTAI, Beijing, China), which was free of folic acid and anaerobically incubated at 37 °C for 48 h. Bacterial growth was determined by measuring absorbance at 600 nm using a Multiskan FC microplate reader (Thermo Scientific). Strains with OD_600_ above 0.2 were deemed to have successful bacterial growth and were identified as potential folate producers.

### 2.2 Evaluation of folate-producing ability

Potential folate producers were further incubated in MRS broth anaerobically for 24 h, and the bacterial cultures were collected and centrifuged at 5,000 × g for 15 min at 4 °C. Bacterial culture supernatants were reserved for folate quantification by microbiological methods as previously reported (16). Total folate was quantified by microbiological methods as previously described. Briefly, 50 μL of bacterial culture was filtered through a 0.2 μm filter and then added to a 96-well plate (BioTAI, Beijing, China) loaded with Folic Acid Assay Broth. The plate was internally coated with freeze-dried *Lacticaseibacillus rhamnosus* ATCC 12126, an auxotrophic strain for folate. Various concentrations of the folate standard, ranging from 16 ng/mL to 128 ng/mL, were prepared for standardization. After incubation in the dark for 48 h, growth was determined by measuring OD_600_. The concentration of the bacterial culture was calculated against standard concentrations. The bacterial cultures that exceeded the standard range were further diluted accordingly and measured again.

The isolates with efficient folate-producing ability were further identified by 16S ribosomal RNA gene (16S rRNA) sequencing, and phylogenetic trees were constructed using the MEGA 5.1 package (18).

### 2.3 Determination of folate and folate-derived metabolites in fermented milk by LC–MS/MS

LC–MS/MS was used to determine folate and its derived metabolites in fermented milk as previously reported with modification (12, 16, 19). The samples were initially spiked with three mixed internal standards ([13C5]-Folic Acid (SHANGHAI ZZBIO CO., LTD), deuterated labeled Folinic acid (Toronto Research Chemicals), and calcium d,l-5-methyltetrahydrofolate (SHANGHAI ZZBIO CO., LTD)). To extract and prepare the folates for analysis, the samples were treated with papain and *α*-amylase to release matrix-bound folates, followed by the addition of rat serum to deconjugate polyglutamated folates into monoglutamate. Then, the mixtures were centrifuged at 10,000 r/min and 4 °C for 5 min. The supernatant was methanol-precipitated, filtered, and dried via nitrogen-blow and reconstituted for analysis by LC–MS/MS.

The analysis was conducted using an LC–MS/MS system with a Kinetex C18 column (2.6 μm, 2.6 mm × 3 mm, MS-062) and a 4,500 triple quadrupole mass spectrometer (AB SCIEX) equipped with an ESI source. The chromatographic conditions were as follows: column temperature 40 °C, auto-sampler temperature 4 °C, flow rate 0.4 mL/min, injection volume 10 μL, and mobile phase consisting of 0.1% formic acid in water (A) and 0.1% formic acid in methanol (B) with a gradient elution program. The mass spectrometer was operated in positive ion mode with multiple reaction monitoring (MRM) scanning, and the key parameters included collision gas (CAD) 9, curtain gas (CUR) 25, nebulizer gas (Gas1) 60.0, auxiliary gas (Gas2) 60.0, spray current (IS) 5,500 V, and atomization temperature (TEM) 550 °C.

Standard curves were prepared for quantification: mixed folate standard solutions (folate, 6S-5-methyltetrahydrofolate, 5-formyltetrahydrofolate) were serially diluted to prepare standards. Additionally, the standard curves for N-(4-aminobenzoyl)-L-glutamic acid, Mefox, and tetrahydrofolate were prepared through stepwise dilution. All standards were from Sigma-Aldrich. Quality control (QC) solutions were prepared using QC reserve solutions to ensure analytical reliability.

### 2.4 Animal grouping and treatment

All animal procedures were performed in accordance with the Guidelines for Care and Use of Animals of Kezhuo Medical Laboratory Testing Co., Ltd. (Shenzhen, China) and approved by the Animal Ethics Committee of Kezhuo Medical Laboratory Testing Co., Ltd. (Shenzhen, China). Male C57BL/6 mice (5–6 weeks old, ~18 g, *n* = 36) were used in this study. After 1 week of acclimatization, the mice were randomly assigned to a normal diet group (Nd, *n* = 6) or a folate-deficient diet group (*n* = 30). Mice in the Nd group were fed a standard chow diet, whereas mice in the folate-deficient group were fed a folate-deficient diet (AIN-93; Ready Dietech). After 1 month of dietary intervention, 10 μL of blood was collected from the tail vein, and serum folate concentrations were measured by microbiological assay to confirm successful establishment of the folate-deficient mouse model. Subsequently, mice were treated with a candidate probiotic cocktail. The candidate probiotic cocktail consisted of seven isolated bacterial strains (*Lactiplantibacillus plantarum* FL001, *Lactiplantibacillus plantarum* Ausa002, *Lactiplantibacillus plantarum* Ausa004, *Bifidobacterium longum* subsp. longum FL006, *Bifidobacterium breve* FL008, *Heyndrickxia coagulans* AUSA001, and *Bifidobacterium animalis* subsp. lactis BB100) and was prepared according to the detailed procedures described in the Supplementary methods. Each strain was activated separately, fermented under strain-specific growth conditions, harvested by centrifuge, washed with sterile PBS, and lyophilized before being mixed at the designated viable-cell ratio for administration.

Mice fed the folate-deficient diet were further subdivided into the following groups: folate-deficient diet control (FA_d), high-dose candidate probiotic cocktail (Pro_H), medium-dose candidate probiotic cocktail (Pro_M), low-dose candidate probiotic cocktail (Pro_L), and synthetic folic acid positive control (FA). Mice in the Nd and FA_d groups were orally gavaged with 200 μL/day of sterile 1 × PBS. Mice in the Pro_H group received 200 μL/day of candidate probiotic cocktail suspension at a concentration of 1 × 10^10^ CFU/mL, whereas mice in the Pro_M and Pro_L groups received candidate probiotic suspensions at concentrations of 1 × 10^9^ CFU/mL and 1 × 10^8^ CFU/mL, respectively. Mice in the FA group were orally gavaged with 200 μL/day of folic acid solution (9.125 μg/mL), corresponding to a daily dose of 73 μg folic acid per kilogram of body weight.

Fecal and blood samples were collected 1 day after the completion of the 6-week candidate probiotic treatment. Quantitative PCR (qPCR) using primers specific to the administered bacterial species was performed to evaluate the transient persistence of probiotic strains. Serum folate and homocysteine (Hcy) levels were determined using chemiluminescent immunoassays (Folic acid Assay Kit, Tailored Medical Ltd).

### 2.5 Quantitative real-time PCR (qPCR) detection of candidate probiotic species

Quantitative real-time PCR (qPCR) was performed to determine the relative abundance of the administered candidate probiotic species in mouse fecal samples collected 1 day after the completion of the 6-week oral gavage treatment. Species-specific primers targeting the administered bacterial strains were adapted from previous publications (Supplementary Table 1). Relative fold changes in target bacterial abundance were calculated via the 2^–∆∆Ct^ comparative threshold method.

### 2.6 Gut microbiota sequencing and bioinformatics

Extraction of fecal DNA and sequencing were performed using previously described methods (17). Bacterial genomic DNA was extracted from fecal samples using MagPure Stool DNA KF Kit B (MAGEN, Guangzhou, China) according to the manufacturer’s protocol. The extracted DNA was used as the template to amplify the V3–V4 variable region of 16S rDNA of bacteria by forward and reverse PCR degenerate primers F and R (338F: ACTCCTACGGGAGGCAGCAG, 806R: GGACTACHVGGGTWTCTAAT). Paired-end (PE300) sequencing reads were generated on the DNBSEQ-G400 platform (BGI-Shenzhen, China). QIIME 2 pipeline (v1.8) was used to process the raw sequencing files.^1^ High-quality paired-end reads were combined to tags with an average read length of 252 bp using FLASH. The tags were then clustered to operational taxonomic units (OTUs) using USEARCH (v. 7.0.1090) at 97% sequence similarity (20). OTU representative sequences were aligned against the database for taxonomic annotation by RDP Classifier (v2.2) software (21). Alpha diversity was mainly measured using Sobs, Chao, ACE, and the Shannon index in mothur (v.1.31.2) (22). Community dissimilarity was analyzed by Principal component analysis (PCA). Weighted UniFrac distance between groups was caculated, and permutational multivariate analysis of variance (PERMANOVA) using the adonis2 function in the vegan R package (2024.12.1) was used to test for significant differences among groups (23). Differential abundance analysis of bacterial taxa was conducted using the analysis of compositions of microbiomes with bias correction 2 (ANCOM-BC2) algorithm via the q2-composition plugin in QIIME 2. The Holm–Bonferroni method was applied to adjust *p*-values, and taxa were considered significantly different at a threshold of adjusted *p* < 0.05.

### 2.7 Statistical analysis

All statistical analyses were performed using GraphPad Prism 9 (GraphPad Software, Inc.). Statistical differences between experimental groups were evaluated by Student’s t-tests and one-way analysis of variance (ANOVA) with a Dunnett’s *post hoc* test for multiple comparisons. All data were expressed as means ± standard deviations (SDs). *p*-values <0.05 were considered statistically significant.

## 3 Results

### 3.1 Isolation of folate-producing candidate probiotic strains

More than 1,000 bacterial isolates were obtained through high-throughput cultivation and screening. Thirty-one bacterial strains, representing the genera *Bacillus*, *Streptococcus*, *Lacticaseibacillus*, *Lactiplantibacillus*, *Bifidobacterium*, and *Heyndrickxia*, were identified via 16S rRNA gene sequencing (Supplementary Figure 1). Their folate-producing capabilities were evaluated using a microbiological assay. The results identified eight strains capable of producing folate at levels exceeding 100 ng/mL (Table 1). These high-producing isolates were classified as *Bacillus cereus*, *Streptococcus thermophilus*, *Bifidobacterium longum* subsp. longum, *Lactiplantibacillus plantarum*, and *Heyndrickxia coagulans*.

**Table 1:** Total folate production of the isolated candidate probiotic strains quantified by microbiological assay.

No.	Isolated candidate probiotic strains	Sources	Supernatant folate (ng/mL)
1	*Bacillus cereus* AF003	Fermented food	98.7 ± 7.8
2	*Bacillus cereus* AUSA008	Human stool	129.5 ± 11.5
3	*Streptococcus thermophilus* AF006	Fermented food	127.0 ± 20.8
4	*Streptococcus thermophilus* AF007	Fermented food	84.3 ± 13.6
5	*Lacticaseibacillus paracasei* AF008	Fermented food	0.6 ± 0.2
6	*Bifidobacterium breve* FL008	Human stool	62.7 ± 19.9
7	*Bifidobacterium breve* AUSA012	Human stool	51.2 ± 24.0
8	*Bifidobacterium adolescentis* AUSA013	Human stool	62.7 ± 4.4
9	*Bifidobacterium longum* subsp. *longum* AUSA015	Human stool	136.7 ± 15.1
10	*Bifidobacterium longum* subsp. *longum* AUSA016	Human stool	72.1 ± 15.0
11	*Bifidobacterium longum* subsp. *longum* FL006	Human stool	114.0 ± 24.0
12	*Lactiplantibacillus plantarum* AUSA002	Fermented food	113.4 ± 13.2
13	*Lactiplantibacillus plantarum* AUSA004	Human stool	113.0 ± 12.8
14	*Lactiplantibacillus plantarum* FL001	Human stool	131.0 ± 19.7
15	*Lactiplantibacillus plantarum* AUSA019	Human stool	89.6 ± 24.7
16	*Lacticaseibacillus rhamnosus* AF009	Human stool	1.4 ± 0.6
17	*Lacticaseibacillus rhamnosus* AUSA011	Human stool	0.8 ± 0.2
18	*Lacticaseibacillus rhamnosus* AUSA020	Fermented food	0.7 ± 0.5
19	*Limosilactobacillus fermentum* AOSA CECT100	Fermented food	2.5 ± 1.3
20	*Bifidobacterium animalis* subsp. *lactis* AOSA BB100	Fermented food	3.6 ± 1.7
21	*Bifidobacterium animalis* subsp. *lactis* AOSA BB100A	Fermented food	3.6 ± 1.1
22	*Bifidobacterium animalis* subsp. *lactis* AOSA BB100B	Fermented food	2.5 ± 1.0
23	*Bifidobacterium animalis* subsp. *lactis* AOSA BB100C	Fermented food	5.8 ± 1.8
24	*Bifidobacterium bifidum* AOSA R100	Human stool	68.6 ± 16.9
25	*Lactiplantibacillus acidophilus* AOSA NCFM100	Fermented food	1.1 ± 0.4
26	*Lactiplantibacillus acidophilus* AOSA NCFM100A	Fermented food	1.1 ± 0.3
27	*Heyndrickxia coagulans* FL009	Fermented food	79.6 ± 12.2
28	*Heyndrickxia coagulans* FL010	Fermented food	53.9 ± 9.2
29	*Heyndrickxia coagulans* FL011	Fermented food	43.4 ± 20.5
30	*Heyndrickxia coagulans* AUSA001	Human stool	114.4 ± 26.8
31	*Lactiplantibacillus delbrueckii* subsp. *bulgaricus* FL012	Fermented food	1.9 ± 0.5

### 3.2 Strain-specific folate biosynthesis pathways and potential of microbial cross-feeding

**Figure 1:**
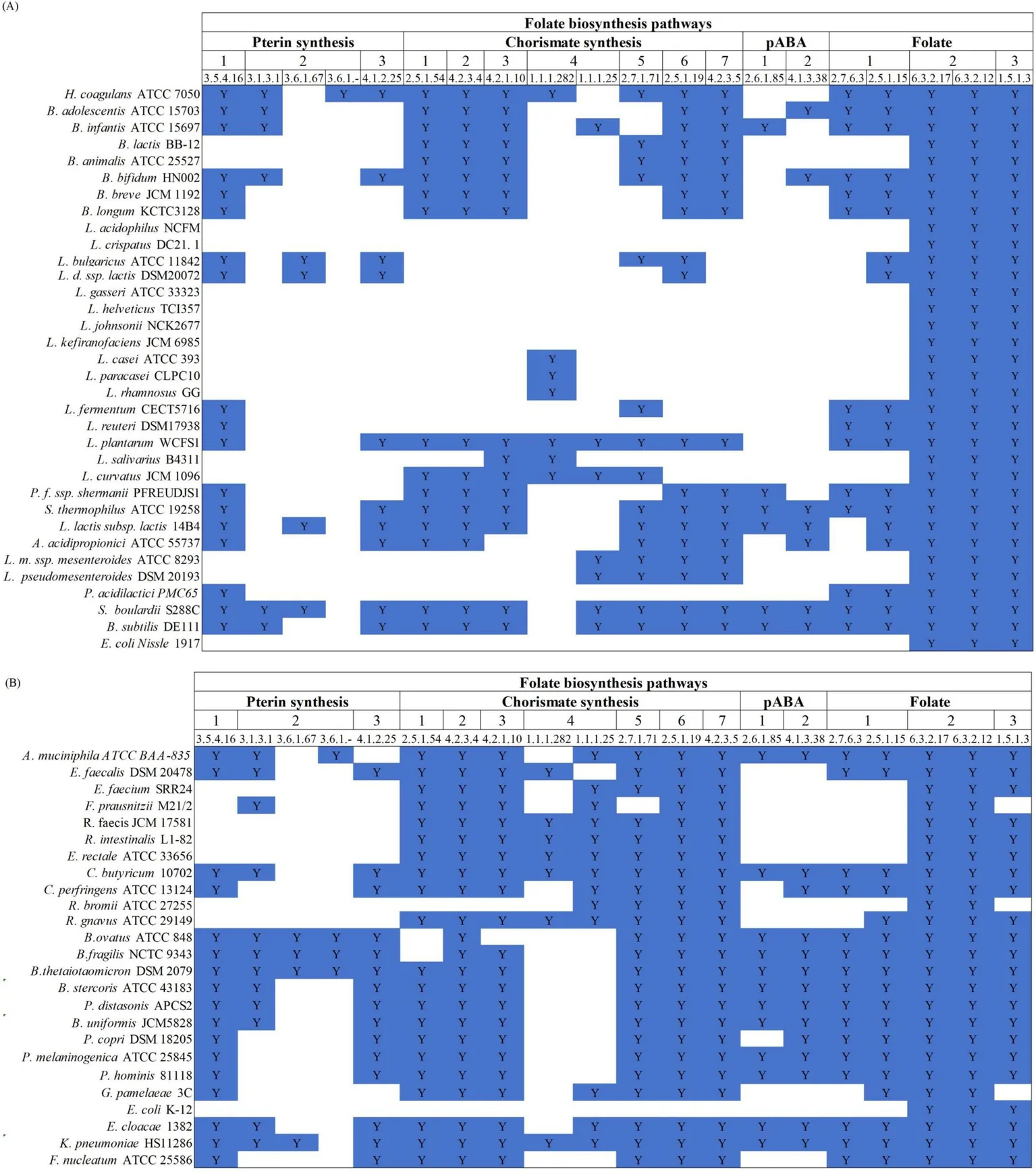
Folate *de novo* synthesis pathways analysis in **(A)** candidate probiotic strains and **(B)** common gut commensals. Y: presence of the corresponding gene. The genome sequences of the candidate probiotic strains and gut commensals were downloaded from the NCBI database (https://www.ncbi.nlm.nih.gov/). KofamKOALA (https://www.genome.jp/tools/kofamkoala/) was used to assign K numbers to the sequences by HMMER/HMMSEARCH against KOfam [a customized HMM database of KEGG Orthologs (KOs)]. The predicted KOs associated with chorismate synthesis, pABA synthesis, pterin synthesis, and folate synthesis were mapped to folate biosynthesis pathways by the KEGG mapper (https://www.kegg.jp/kegg/mapper).

Whole-genome analysis and pathway reconstruction of 34 commonly used candidate probiotic strains revealed substantial heterogeneity in folate biosynthesis gene content among these strains (Figure 1). Generally, species demonstrating relatively high folate-producing capacity when cultured in standard MRS broth (e.g., *B. cereus*, *L. plantarum*, *S. thermophilus*, and *H. coagulans*) (Table 1) possessed more comprehensive upstream folate biosynthesis pathways (Figure 1A). In contrast, strains exhibiting minimal folate production in MRS (e.g., *L. paracasei*, *L. rhamnosus*, *L. acidophilus*, and *B. animalis* subsp. *lactis*) (Table 1) typically lacked key genes involved in pterin, chorismate, or para-aminobenzoic acid (pABA) biosynthesis (Figure 1A).

Notably, while all 34 analyzed strains harbored the downstream segment of the folate biosynthesis pathway (converting dihydropteroate to THF-polyglutamate; including enzymes annotated as EC 6.3.2.17, EC 6.3.2.12, and EC 1.5.1.3), none of the candidate probiotic strains possessed a complete *de novo* pathway. Specifically, all analyzed strains lacked a complete set of genes required for the upstream biosynthesis of either pterin, chorismate, or pABA (Figure 1A). In contrast, genomic analysis of 25 representative gut commensals across six phyla (Firmicutes, Bacteroidetes, Actinobacteria, Proteobacteria, Verrucomicrobia, and Fusobacteria) revealed that several commensals, particularly members of *Bacteroides* and *Prevotella*, harbored complete pterin- and pABA-biosynthesis gene clusters (Figure 1B). These comparative results suggest that folate production by these candidate probiotics is genetically incomplete and inherently dependent on external precursor sources supplied either by the host diet or through cross-feeding from native gut commensals.

### 3.3 Characterization of folate and folate-derived metabolite production in candidate probiotic-fermented milk

**Figure 2:**
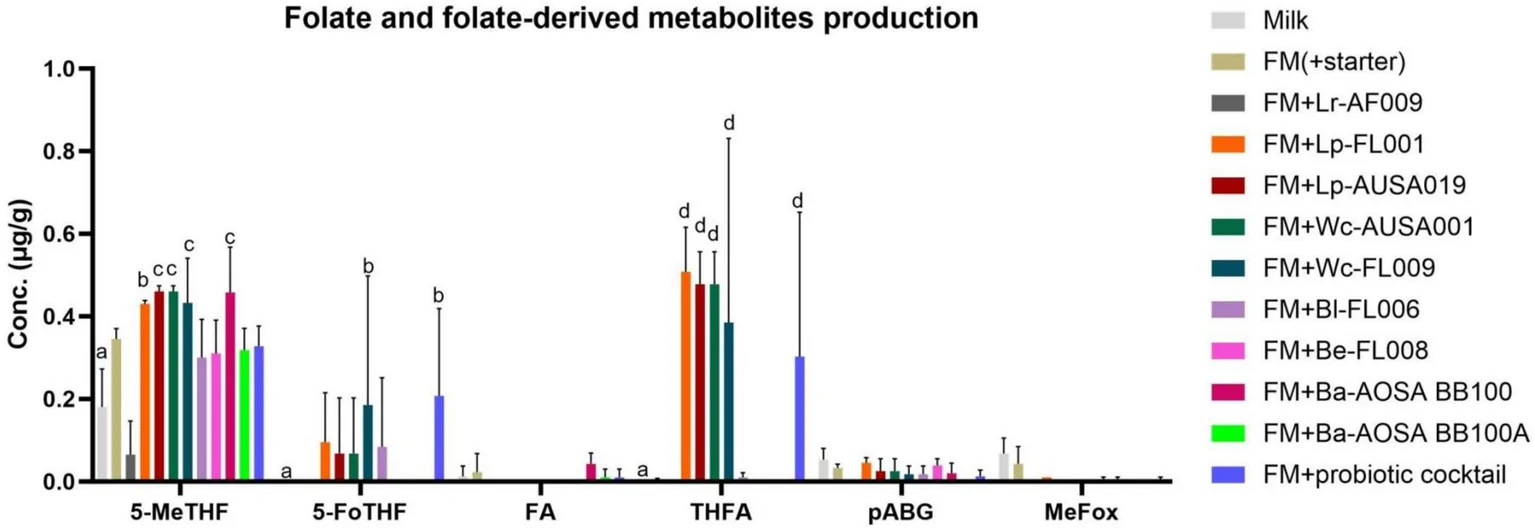
Folate and folate-derived metabolite production in milk fermented by candidate probiotic strains. Milk was fermented with starter (*Streptococcus thermophilus* AF006 and *Lactiplantibacillus delbrueckii* subsp. *bulgaricus* FL006) and candidate probiotic strains for 18 h; folate and folate-derived metabolites produced were determined by LC-MS/MS. 5-MeTHF, 5-methyltetrahydrofolate; 5-FoTHF, 5-formyltetrahydrofolate; FA, folic acid; THF, tetrahydrofolate; pABG, P-aminobenzoyl glutamate; MeFox, (6RS)-MeFox. Statistical differences were determined by one-way analysis of variance (ANOVA) with a Dunnett’s test for multiple comparisons.

Analysis of folate and folate-derived metabolites in candidate probiotic-fermented milk showed that the main folate forms produced by the candidate probiotic strains were the biologically active vitamers 5-methyltetrahydrofolate (5-MeTHF) and tetrahydrofolate (THF), while folate-producing capacity varied considerably among strains (Figure 2). *L. plantarum* species, *H. coagulans* species, *B. animalis* subsp. *lactis*, and the fermentation starter (*S. thermophilus* and *L. delbrueckii* subsp. *bulgaricus*) significantly increased the amount of 5-MeTHF in fermented milk. Only *L. plantarum* species and *H. coagulans* significantly increased THF concentration. In contrast, it showed that the base level of 5-MeTHF in the fermented milk (with starter) decreased after the addition of *L. rhamnosus*. This is consistent with the folate synthesis pathway analysis, which showed *L. rhamnosus* lacks the *de novo* folate synthesis pathway and has to scavenge folate from the external environment. Interestingly, *B. animalis* subsp. *lactis* genome analysis showed a lack of the de novo folate synthesis pathway but showed a relatively high ability to produce 5-MeTHF, indicating that the milk fermented with starter contains the precursor of folate de novo synthesis.

### 3.4 Effects of a candidate probiotic cocktail on mice’s folate and homocysteine metabolism

**Figure 3:**
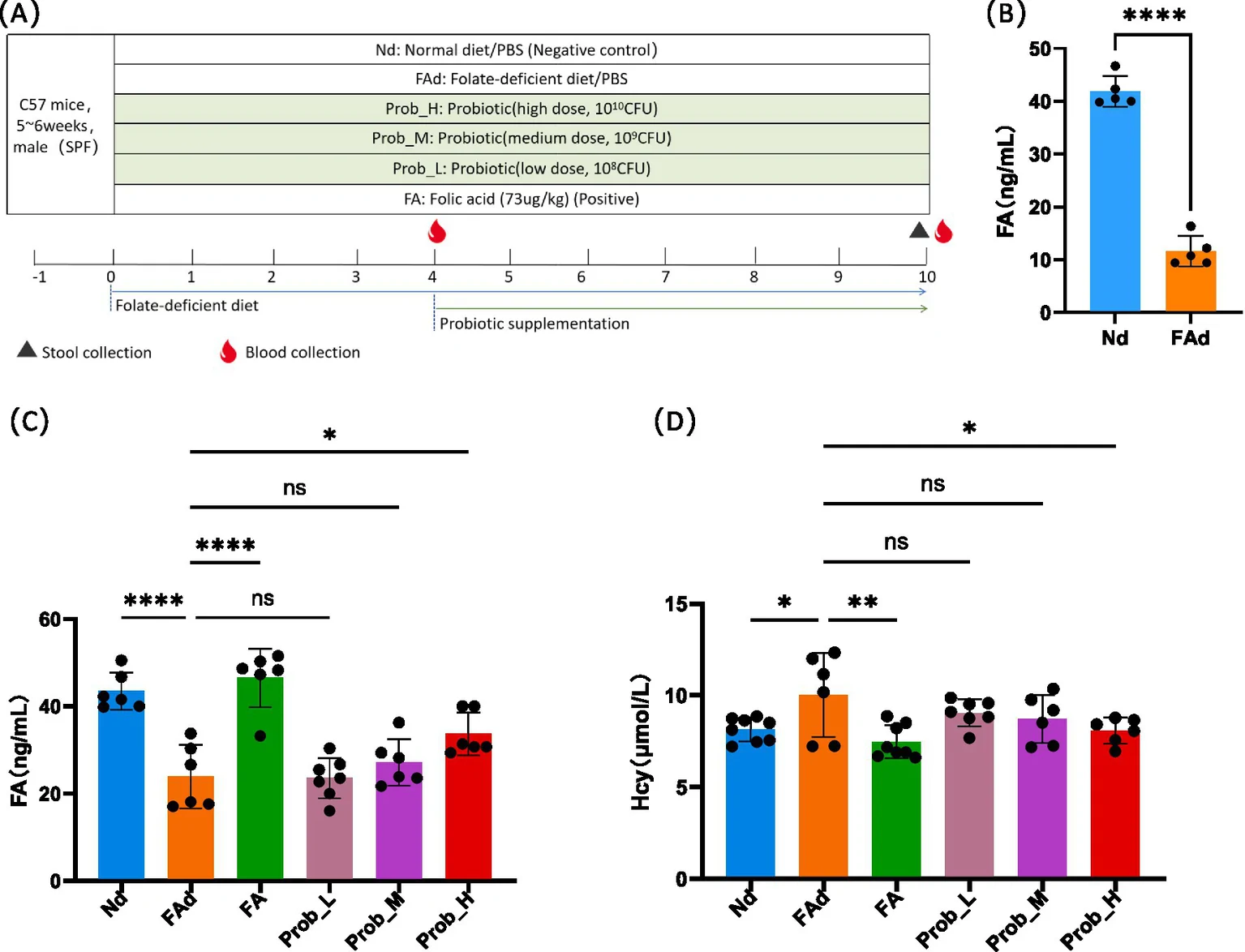
Candidate probiotic cocktail administration improves folate status and reduces homocysteine levels in folate-deficient mice. **(A)** Mouse group and intervention design. Nd, normal diet/PBS (negative control); FAd, folate-deficient diet/PBS; Prob_H, candidate probiotic (high dose, 10^10^ CFU); Prob_M, candidate probiotic (medium dose, 10^9^ CFU); Prob_L, candidate probiotic (low dose, 10^8^ CFU); FA, folic acid (73 μg/kg) (positive). **(B)** Mice folate levels determined by microbiological assays after 1 month on a folate-deficient diet. **(C)** Mice folate and **(D)** homocysteine levels determined by chemiluminescence assays after 6 weeks of candidate probiotic cocktail administration. Each dot represents an individual mouse. Data are presented as mean ± SD. Statistical differences between experimental groups were evaluated by Student’s *t*-tests and one-way analysis of variance (ANOVA) with a Dunnett’s test for multiple comparisons. *p < 0.05, **p < 0.01, and ***p < 0.001.

A folate-deficient diet was used to establish a folate-deficient mouse model (Figure 3A). Mice’s body weight was recorded during the intervention. Neither the folate-deficient diet nor the candidate probiotic cocktail treatment significantly affected body weight (Supplementary Figure 2).

After 1 month on a folate-deficient diet, blood was collected from the tail veins of five mice. Serum folate levels were determined using a microbiological assay. The results demonstrated that serum folate levels were significantly reduced in the folate-deficient group compared to the normal diet group (Figure 3B), indicating the successful establishment of a folate-deficient mouse model after 1 month of dietary intervention.

After 6 weeks of candidate probiotic administration, blood was collected from the orbital sinus. Serum folate and homocysteine (Hcy) levels were quantified using chemiluminescent immunoassays. The results showed that serum folate levels were significantly elevated in the high-dose candidate probiotic group (*p* < 0.01), while a non-significant increasing trend was observed in the medium-dose group (*p* > 0.05) (Figure 3C). Correspondingly, serum Hcy levels were significantly increased in the folate-deficient diet group. Administration of synthetic folate significantly decreased serum Hcy levels (*p* < 0.01) (Figure 3D). Similarly, a significant reduction in serum Hcy was observed in the high-dose candidate probiotic group (*p* < 0.05), whereas no significant changes were detected in the medium- and low-dose groups.

### 3.5 Fecal detection and transient persistence of administered candidate probiotics in mice

**Figure 4:**
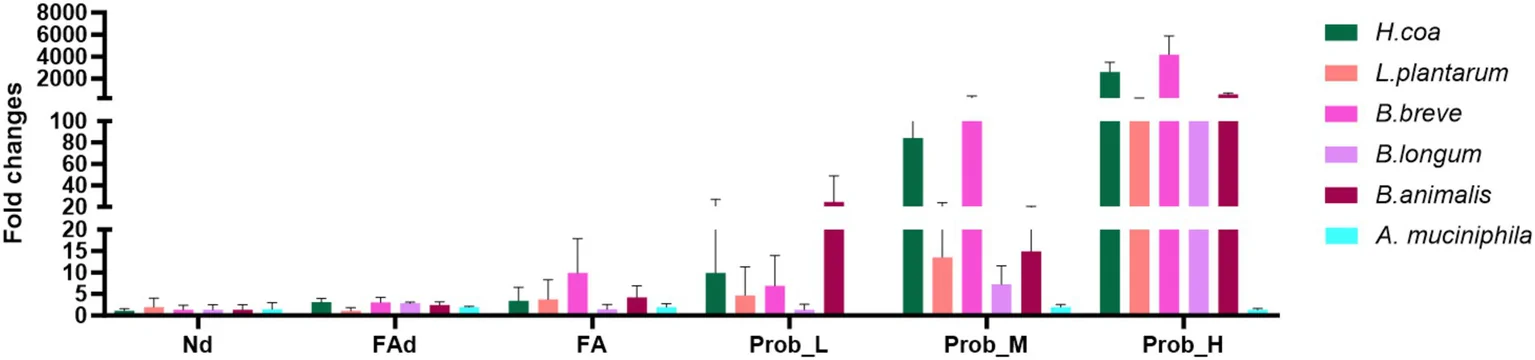
qPCR analysis of the relative abundance of candidate probiotic strains in mouse feces after 6-week candidate probiotic cocktail administration. The relative abundance of administered strains, including *L. plantarum* strains, *B. longum* subsp. *longum* FL006, *B. breve* FL008, *H. coagulans* AUSA001, and *B. animalis* subsp. *lactis* BB100, in mice feces was quantified by qPCR. The relative changes in bacterial abundance were calculated using the 2^∆∆Ct method.

The fecal qPCR results demonstrated that the relative abundance of candidate probiotic species increased in a dose-dependent manner in the mice’s gut after administration (Figure 4). The most significant increase in relative abundance was observed in the high-dose group, reaching a maximum increase of more than 4,000-fold. In contrast, the increase in the low-dose group was limited to less than 100-fold (Figure 4). At the species level, differences in fecal recovery among the administered bacterial species were also observed. In the high-dose group, *H. coagulans* and *B. breve* showed a 2588.4 ± 911.4-fold and 4176.0 ± 1706.9-fold increase in relative DNA abundance, respectively, while *L. plantarum*, *B. longum*, and *B. animalis* exhibited increases of 202.8 ± 39.2, 120.7 ± 15.8-fold, and 561.0 ± 137.9-fold, respectively. Similar patterns were observed in the low- and medium-dose groups.

### 3.6 Effects on mice gut microbiota composition

Analysis of alpha diversity indices revealed no statistically significant differences in Sobs, Chao, ACE, and Shannon indices among the groups following feeding with a folate-deficient diet or potential candidate probiotic cocktail treatment (Supplementary Figure 3).

**Figure 5:**
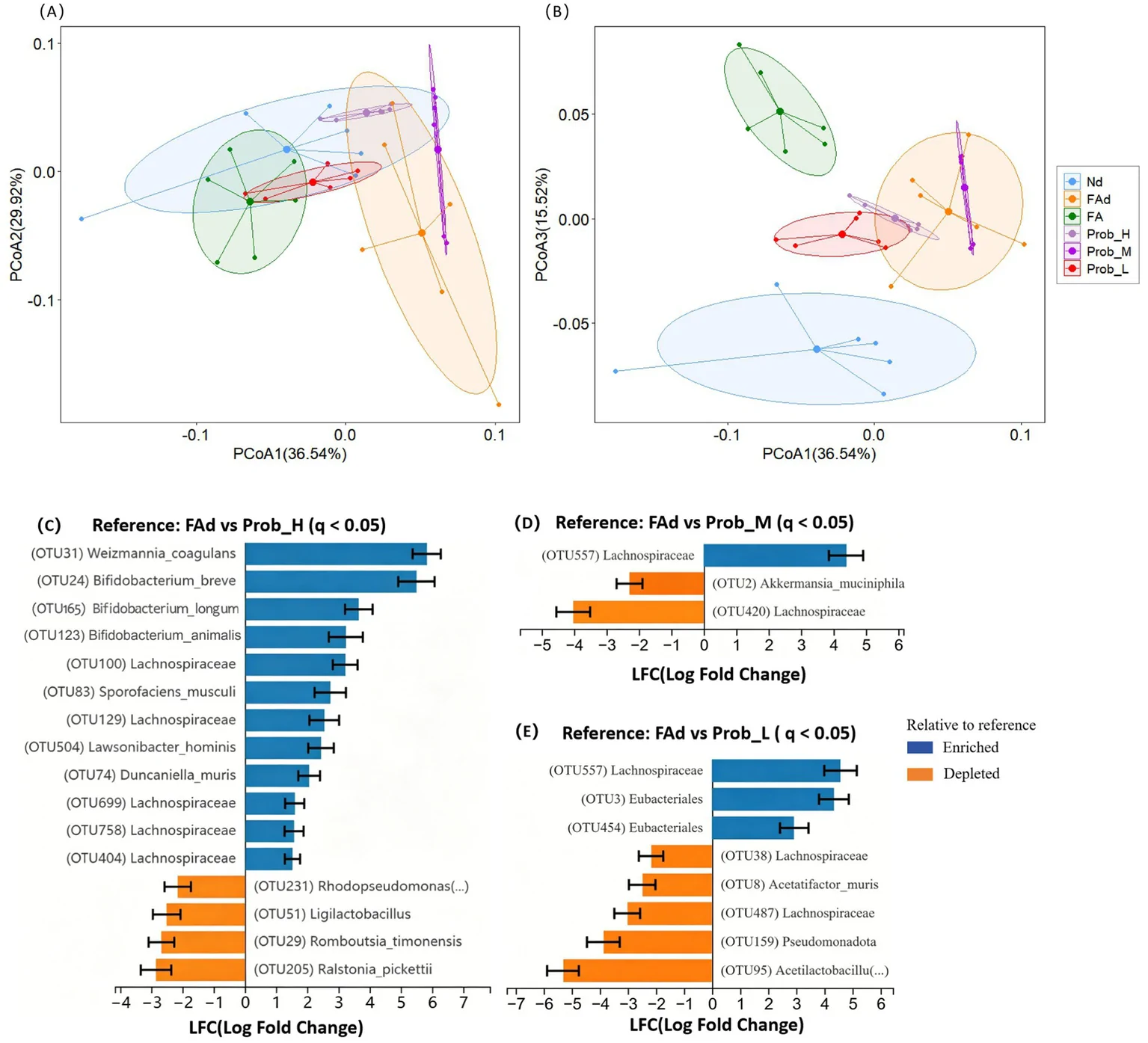
Effects of the candidate probiotic cocktail on gut microbiota composition in mice. Principal coordinate analysis (PCoA) of gut microbial communities based on weighted UniFrac distances. **(A)** Ordination plot of the first and second principal coordinates (PCoA1 and PCoA2). **(B)** Ordination plot of the first and third principal coordinates (PCoA1 and PCoA3). Each point represents the microbiota profile of an individual fecal sample, colored by treatment group as indicated in the legend. Differences in microbiota community structure among groups were assessed using permutational multivariate analysis of variance (PERMANOVA) with 999 permutations on the Weighted UniFrac distance matrix. Nd vs. FAd: *R*^2^ = 0.1368, *p* = 0.011; Nd vs. FA: *R*^2^ = 0.1296, *p* = 0.021; Nd vs. Prob_H: *R*^2^ = 0.0706, *p* = 0.256; Nd vs. Prob_M: *R*^2^ = 0.1045, *p* = 0.056; Nd vs. Prob_L: *R*^2^ = 0.1018, *p* = 0.062. **(C–E)** Differential taxon abundance. ANCOM-BC2 analysis at the OTU level identified differentially abundant taxa between the FAd group and the Prob_H **(C)**, Prob_M **(D)**, and Prob_L **(E)** groups. Significance was determined using Holm–Bonferroni-adjusted *p*-values (adjusted *p* < 0.05).

Principal coordinate analysis (PCoA) was performed to visualize overall gut microbial community composition across different groups (Figures 5A,B). PERMANOVA analysis using the adonis2 function based on weighted UniFrac distances revealed that a folate-deficient diet significantly altered gut microbiota composition compared with the normal diet group (Nd vs. FAd, *R*^2^ = 0.1368, *p* = 0.011). Supplementation with synthetic folic acid also resulted in significant changes when compared with the normal diet (Nd vs. FA: *R*^2^ = 0.1296, *p* = 0.021). In contrast, high, medium, and low doses of the candidate probiotic cocktail showed no statistically significant compositional differences compared with the normal diet group (Nd vs. Prob_H: *R*^2^ = 0.0706, *p* = 0.256; Nd vs. Prob_M: *R*^2^ = 0.1045, *p* = 0.056; Nd vs. Prob_L: *R*^2^ = 0.1018, *p* = 0.062). Moreover, the high-dose probiotic group exhibited no significant separation from the folate-deficient control (FAd vs. Prob_H: *R*^2^ = 0.0840, *p* = 0.147).

ANCOM-BC2 analysis at the OTU level was used to identify taxa that were differentially abundant following administration of the candidate probiotic cocktail (Figures 5C–E). The results demonstrated that the taxa assigned to the species of the supplemented candidate probiotics, including *Heyndrickxia coagulans*, *Bifidobacterium breve*, *Bifidobacterium longum*, and *Bifidobacterium animalis*, were significantly enriched in mice receiving the high-dose treatment, indicating transient persistence of these administered species (Figure 5C). Moreover, several taxa assigned to the *Lachnospiraceae* and *Oscillospiraceae* families, including *Sporofaciens musculi* and *Lawsonibacter hominis*, were enriched in the high-dose candidate probiotic group. In contrast, taxa assigned to potentially opportunistic pathogens such as *Ralstonia pickettii* and *Romboutsia timonensis* were significantly depleted following candidate probiotic treatment. However, this enrichment of the taxa assigned to candidate probiotics was not observed in the medium-dose (Figure 5D) or low-dose (Figure 5E) groups.

## 4 Discussion

This study demonstrates that selectively screened folate-producing candidate probiotic strains can synthesize bioactive folate, particularly 5-methyltetrahydrofolate (5-MeTHF), and that supplementation with these strains improves host folate status and homocysteine (Hcy) metabolism *in vivo*. These findings provide both mechanistic and functional evidence supporting the role of candidate probiotics in modulating micronutrient status and Hcy metabolism.

We characterized the folate-producing capacity of the isolated strains by measuring total folate using microbiological methods. Consistent with previous studies (9, 11, 13, 15), isolated candidate probiotic strains from various bacterial species produced variable amounts of folate in MRS broth. Genome-based folate pathway reconstruction, however, revealed that *de novo* folate biosynthesis is generally incomplete across these candidate probiotic strains. A common limitation was the absence of genes required for para-aminobenzoic acid (pABA) synthesis, a key precursor for folate production. This metabolic deficiency indicates that these candidate probiotic strains rely on exogenous precursors supplied by the MRS broth to support folate production. This observation may further account for discrepancies in folate levels reported across studies, as the composition of the culture media used often varies between investigations (24).

In contrast, many prevalent gut commensals, such as *Bacteroides* and *Prevotella*, encoded more complete folate biosynthesis pathways, including complete pterin- and pABA-biosynthesis gene clusters. This complementarity supports a potential cross-feeding model in which probiotics may rely on microbiota-derived precursors (e.g., pABA) to support folate formation in the gut. Such metabolic cooperation is consistent with previous systematic genome-based assessments of B-vitamin biosynthesis, which suggest cooperation among gut microbes (25).

Furthermore, we characterized the molecular forms of folate produced by screened candidate probiotics using LC–MS/MS. The results confirmed that the folate produced by the isolated candidate probiotic strains in fermented milk is largely in the physiologically active form, 5-methyltetrahydrofolate (5-MeTHF). Interestingly, genome analysis revealed that *B. animalis* species encoded only a downstream segment of the folate biosynthesis pathway (from dihydropteroate to THF-polyglutamate; including enzymes annotated as EC 6.3.2.17, EC 6.3.2.12, and EC 1.5.1.3). However, isolated *B. animalis* AOSA BB100 generated substantial amounts of 5-MeTHF and THF during milk fermentation. This discrepancy highlights two key points: (i) the presence/absence of canonical genes does not necessarily translate into functional folate production under specific growth conditions and (ii) standard laboratory media may not reflect the precursor availability, redox state, or metabolic cues present in food matrices or the gut. Thus, functional validation under biologically relevant conditions remains essential alongside *in silico* predictions.

5-MeTHF is the predominant bioactive form of folate circulating in humans and can be utilized directly in one-carbon metabolism without requiring extensive enzymatic conversion (26). In contrast, synthetic folic acid must first be reduced to 5-MeTHF, a process that depends in part on methylenetetrahydrofolate reductase (MTHFR) activity. Reduced MTHFR function associated with common genetic variants (e.g., the 677C > T polymorphism) can compromise this conversion efficiency and has been linked to elevated homocysteine and impaired folate status in susceptible individuals (27). Collectively, these considerations suggest that candidate probiotic-mediated delivery of 5-MeTHF may offer advantages over folic acid supplementation, providing a potentially more efficient and physiologically relevant strategy to improve folate status *in vivo*.

Therefore, we designed an *in vivo* candidate probiotic cocktail by prioritizing high *in vitro* folate yields and formulating a taxonomically diverse consortium (*L. plantarum*, *H. coagulans*, *B. breve*, *B. longum*, and *B. animalis*) with the aim of combining strains that possess robust downstream conversion capabilities alongside those with broader upstream genetic potential.

Indeed, our animal experiment demonstrated that supplementation with the candidate probiotic cocktail improved folate levels and reduced Hcy, but these effects were significant only in the high-dose group. This dose-dependence aligns with our qPCR results, which show that only high-dose probiotic administration significantly increased the relative abundance of the supplemented species in feces. Probiotics are defined as live microorganisms that, when administered in adequate amounts, confer a health benefit on the host (28). Similarly, our data suggest a threshold effect: a sufficient inoculum is required to achieve meaningful gut exposure (or transient persistence) and thereby elicit measurable improvements in folate availability and downstream one-carbon metabolism, as reflected by reduced Hcy.

In addition to their direct physiological effects, probiotic supplementation has emerged as a promising strategy to modulate the gut microbiota (29). In the present study, supplementation with the candidate probiotic cocktail or feeding a folate-deficient diet did not significantly affect alpha diversity indices (including Sobs, Chao, ACE, and Shannon indices). However, beta-diversity analysis revealed that the folate-deficient diet significantly altered the gut microbiota composition when compared with the normal diet group. Supplementation with synthetic folic acid improved the folate level but had no significant effect on the gut microbiota disrupted by a folate-deficient diet. In contrast, the microbial composition of mice receiving the candidate probiotic cocktail, particularly at the high dose, did not differ significantly from that of mice fed a normal diet or a folate-deficient diet. These results suggest the candidate probiotic treatment did not completely restore the gut microbiota dysbiosis caused by the folate-deficient diet but rather induced a trend in the microbial composition toward that of the normal-diet group.

Differential abundance analysis using ANCOM-BC2 further supported a dose-dependent effect of the candidate probiotic cocktail on gut microbial composition. In the high-dose probiotic group, taxa corresponding to the administered candidate probiotics (*Heyndrickxia coagulans*, *Bifidobacterium breve*, *Bifidobacterium longum*, and *Bifidobacterium animalis*) were significantly enriched, suggesting the transient persistence or increased detectability of these supplemented taxa in the gut. In addition, several members of the *Lachnospiraceae* and *Oscillospiraceae* families (e.g., *Sporofaciens musculi* and *Lawsonibacter hominis*), potential short-chain fatty acid producers (30, 31) were enriched after high-dose candidate probiotic treatment, whereas potentially opportunistic taxa (e.g., *Ralstonia pickettii* and *Romboutsia timonensis*) were depleted (32, 33). These findings suggest that high-dose candidate probiotic supplementation may not only introduce folate-producing bacteria directly but also enrich specific microbial taxa associated with a healthy intestinal ecosystem.

Together, the beneficial effects of the candidate probiotic cocktail observed in this study may be attributed to the transient persistence of the administered bacterial species and to the modulation of the gut microbiota through enrichment of potentially beneficial bacterial taxa. Nevertheless, because the present microbiota analysis was based on compositional profiling and the folate level in the colon was not monitored, it cannot directly identify the source of the improvement in serum folate or how it is transferred into host folate pools. Future studies incorporating metagenomics, targeted luminal folate vitamer quantification, isotope-tracing approaches, gnotobiotic models, and rigorous experimental controls, such as heat-inactivated cells or non-folate-producing bacterial controls, will be necessary to distinguish the direct contribution of supplemented strains to broader community-mediated mechanisms.

Notably, findings derived from murine models cannot be directly extrapolated to humans. For instance, it is well-documented that *Bifidobacterium* species are not prominent components of the normal laboratory mouse microbiota and often exhibit poor colonization in this model (34). Our findings in mice corroborate this limitation; among the three *Bifidobacterium* species administered, only *Bifidobacterium breve* demonstrated relatively high and consistent fecal recovery. Therefore, future clinical trials are warranted to determine the precise effective dosages and evaluate the functional efficacy of these strains in human subjects.

## 5 Conclusion

In summary, this study provides preclinical evidence that selected candidate probiotic strains can produce bioactive 5-MeTHF and that supplementation improves host folate status and lowers homocysteine in a murine model. The observed effects may involve strain-specific metabolic capacity, precursor cross-feeding, and modulation of the gut microbial community under folate-deficient conditions. While these findings highlight the potential of folate-producing probiotics as a microbiota-based strategy to improve folate availability and homocysteine metabolism, further research is necessary to validate their clinical efficacy, physiological advantages over synthetic folic acid, and long-term safety in human populations, including individuals with MTHFR polymorphisms.

## Data Availability

The data presented in the study are deposited in the CNCB-NGDC Genome Sequence Archive (GSA) repository, accession number GSA: CRA048180 (https://ngdc.cncb.ac.cn/gsa).

## Footnotes

1
https://qiime2.org/
