# Supplementary material for: Isolation of folate-producing probiotic candidates and their effects on homocysteine metabolism and gut microbiota composition
Source: Front Nutr. 2026 Aug 20;13:1845105. doi: 10.3389/fnut.2026.1845105 (PMC13539886; doi:10.3389/fnut.2026.1845105)
Supplement: Supplementary file 1 [file Supplementary_file_1.docx]

**Supplementary Methods**

**Preparation of the candidate cocktail**

The candidate cocktail comprised seven folate-producing strains: three Lactiplantibacillus plantarum strains (FL001, AUSA002, and AUSA004), one *Heyndrickxia coagulans* strain (AUSA001), one *Bifidobacterium longum* subsp. longum strain (FL006), and one *Bifidobacterium breve* strain (FL008) and *Bifidobacterium animalis* subsp. lactis BB100.

**Culture and Harvesting:** The three *L. plantarum* strains were cultured in de Man, Rogosa, and Sharpe (MRS) broth at 37 °C under facultative anaerobic conditions. *H. coagulans* AUSA001 was cultured in P01 broth at 40 °C. The three *Bifidobacterium* strains (FL006, FL008 and BB100) were cultured in MRS supplemented with 0.05% L-cysteine at 37 °C under strict anaerobic conditions. Prior to large-scale culture, all strains were activated through three successive subcultures.

For *L. plantarum* strains, the activated strains was then subcultured at a 2% inoculum into 200 mL MRS broth and incubated at 37°C for 16 h with shaking at 160 rpm to obtain the seed culture. Fermentation was performed by inoculating the seed culture into 4 L MRS fermentation medium at a 5% inoculum. The fermentation conditions were 37°C, pH 6.5, and 50 rpm without aeration, as these strains were treated as facultative anaerobes. The cultures were harvested after 16 h when microscopic examination showed uniform cell morphology without contamination and the optical density indicated adequate biomass.

For *H. coagulans* AUSA001, the activated strains was inoculated into 5 mL P01 broth and cultured at 40°C for 16 h with shaking at 160 rpm. The culture was then transferred at a 2% inoculum into 200 ml P01 broth in 500 mL flasks and incubated at 40°C for 16 h with shaking at 160 rpm. Fermentation was carried out in 4 L P01 medium with a 5% inoculum at 37°C, pH 6.5, 150 rpm, and compressed-air aeration at 2 L/min. Glucose solution containing 4 g glucose in 50 mL was supplemented automatically at 8–10 h. The culture was harvested after 44 h, provided that microscopic examination showed uniform morphology and no contamination.

For *Bifidobacterium* strains, the activated strains was inoculated into 5 mL modified MRS broth (supplemented with 0.05% L-cysteine) and cultured at 37°C anaerobically for 16 h. The culture was then subcultured at a 2% inoculum into 200 mL modified MRS broth and incubated anaerobically at 37°C for 16 h to obtain the seed culture. Fermentation was performed in 4 L modified MRS medium with a 5% inoculum at 37°C, pH 6.9, and 100 rpm under nitrogen aeration at 1 L/min. At 12 h, 200 mL glucose solution containing 40 g glucose was added. The culture was harvested after 22 h when the culture showed uniform morphology, no contamination, and the optical density indicated adequate biomass.

**Centrifugation, Washing, and lyophilization:** To eliminate residual folate and other metabolites from the culture medium, the harvested cultures were centrifuged at 4,000 × g for 15 min at 4 °C. The cell pellets were washed twice with sterile phosphate-buffered saline (PBS) and subsequently resuspended in sterile lyoprotectant at a pellet-to-lyoprotectant mass ratio of 1:2. The lyoprotectant contained skim milk powder 12%, sucrose 5%, lactose 3%, trehalose 3%, and fructo-oligosaccharides 5%, and was sterilized at 115°C for 10 min before use. The resuspended bacterial suspension was freeze-dried, collected into sterile food-grade aluminum foil bags, vacuum sealed, and stored at −20°C until use.

**Cocktail Formulation, enumeration and storage:** The the viable cell count of individual lyophilized strain dtermined using the standard spread plate method on modified MRS agar for *Lactobacilli*/*Bifidobacteria* 37 °C and P01 agar for *H. coagulans* after incubation at 40 °C for 48 h under appropriate atmospheric conditions.The results showed the viable of cell count of each lyophilized strain reach appropriately 2×10^11^ CFU/g, then each lyophilized strain were mixed to formulate the candidate cocktail at a ratio of 1:1:1:1:1:1:1 (w/w). The final viable cell count of the candidate cocktail was determined immediately after preparation.The candidate cocktail was stored as a lyophilized powder at -20 °C before use

**Administration Conditions:** The candidate cocktail was prepared freshly on a daily basis by reconstituted the lyophilized powder in appropriate sterile PBS to make the final concentration to 1 × 10¹⁰ CFU/mL,1 × 10^9^ CFU/mL,1 × 10^8^ CFU/mL prior to use.

**Supplementary Tables and Figures**

**Supplementary Table 1. Quantitative PCR (qPCR) primers designed for detection of administered candidate species**

| Targeted candidate species | Primer name | Primer(5’→3’) | Reference |
| --- | --- | --- | --- |
| *Heyndrickxia coagulans* | B.coa-F | CGAACTCGAAGAATATGATGACA | [1] |
|  | B.coa-R | TCATCTTTCGACATGATTTGG |  |
| *Lactiplantibacillus plantarum* | LP-F | CAAGCCTACGCAACTATCAACC | [2] |
|  | LP-R | ACCGTGGGCAAACATTTTATT |  |
| *Bifidobacterium breve* | BiBRE-F | CCGGATGCTCCATCACAC | [3] |
|  | BiBRE-R | ACAAAGTGCCTTGCTCCCT |  |
| *Bifidobacterium longum* | BIL-F | GTTCCCGACGGTCGTAGAG | [4] |
|  | BIL-R | GTGAGTTCCCGGCATAATCC |  |
| *Bifidobacterium animalis* | B.ani-F | GAAAAGGTTGAGAAGGACTTCAACC | [4] |
|  | B.ani-R | GAAGCCCTCACCCTCAG |  |

**Supplementary Figure 1.** Phylogenetic tree of the isolated strains based on 16s rRNA gene sequence.

**Supplementary Figure 2.** The weight change of the mice during intervention. Nd: Normal diet/PBS; FAd: Folate-deficient diet/PBS; Prob_H: candidate probiotic (high dose, 10^10^CFU); Prob_M: candidate probiotic (medium dose, 10^9^CFU); Prob_L: candidate probiotic (low dose, 10^8^CFU); FA: Folic acid (73ug/kg) .

**Supplementary Figure 3.** Comparison of alpha diversity (Sobs, Chao, Shannon index and Chao index) of the gut microbiota based on groups. Alpha diversity was compared across groups using one-way ANNOVA with a Dunnett’s test tests. ns p>0.05.

**Reference**

1.	Chen, Z., et al., *Weizmannia coagulans BCF-01: a novel gastrogenic probiotic for Helicobacter pylori infection control.* Gut Microbes, 2024. **16**(1): p. 2313770.

2.	Shawon, J., et al., *Detection and absolute quantification of Lactiplantibacillus plantarum ATCC 202195 by quantitative real-time PCR.* Microbiology Spectrum, 2024. **12**(1): p. e02711-23.

3.	Wickramasinghe, S., et al., *Bifidobacteria grown on human milk oligosaccharides downregulate the expression of inflammation-related genes in Caco-2 cells.* BMC microbiology, 2015. **15**(1): p. 172.

4.	Matsuki, T., K. Watanabe, and R. Tanaka, *Genus-and species-specific PCR primers for the detection and identification of bifidobacteria.* Current Issues in Intestinal Microbiology, 2003. **4**(2): p. 61-69.
